# Mechanism of Rapid Nuclear Factor-E2-Related Factor 2 (Nrf2) Activation via Membrane-Associated Estrogen Receptors: Roles of NADPH Oxidase 1, Neutral Sphingomyelinase 2 and Epidermal Growth Factor Receptor (EGFR)

**DOI:** 10.3390/antiox8030069

**Published:** 2019-03-18

**Authors:** Tetsuro Ishii, Eiji Warabi

**Affiliations:** Faculty of Medicine, University of Tsukuba, Tsukuba Ibaraki 305-8575, Japan; warabi-e@md.tsukuba.ac.jp

**Keywords:** estrogen, MCF-7, Nrf2, ER-α36, GPER, EGFR, NOX1, neutral sphingomyelinase 2, ceramide, ERK, p38, PKCδ, PKCζ, CK2, Akt, PI3K, phytoestrogen

## Abstract

Membrane-associated estrogen receptors (ER)-α36 and G protein-coupled estrogen receptor (GPER) play important roles in the estrogen’s rapid non-genomic actions including stimulation of cell proliferation. Estrogen via these receptors induces rapid activation of transcription factor nuclear factor-E2-related factor 2 (Nrf2), a master regulator of detoxification and antioxidant systems, playing a key role in the metabolic reprogramming to support cell proliferation. This review highlights the possible mechanism underlying rapid Nrf2 activation via membrane-associated estrogen receptors by estrogen and phytoestrogens. Stimulation of ER-α36-GPER signaling complex rapidly induces Src-mediated transactivation of epidermal growth factor receptor (EGFR) leading to a kinase-mediated signaling cascade. We propose a novel hypothesis that ER-α36-GPER signaling initially induces rapid and temporal activation of NADPH oxidase 1 to generate superoxide, which subsequently activates redox-sensitive neutral sphingomyelinase 2 generating the lipid signaling mediator ceramide. Generation of ceramide is required for Ras activation and ceramide-protein kinase C ζ-casein kinase 2 (CK2) signaling. Notably, CK2 enhances chaperone activity of the Cdc37-Hsp90 complex supporting activation of various signaling kinases including Src, Raf and Akt (protein kinase B). Activation of Nrf2 may be induced by cooperation of two signaling pathways, (i) Nrf2 stabilization by direct phosphorylation by CK2 and (ii) EGFR-Ras-PI 3 kinase (PI3K)-Akt axis which inhibits glycogen synthase kinase 3β leading to enhanced nuclear transport and stability of Nrf2.

## 1. Introduction

**Figure 1:**
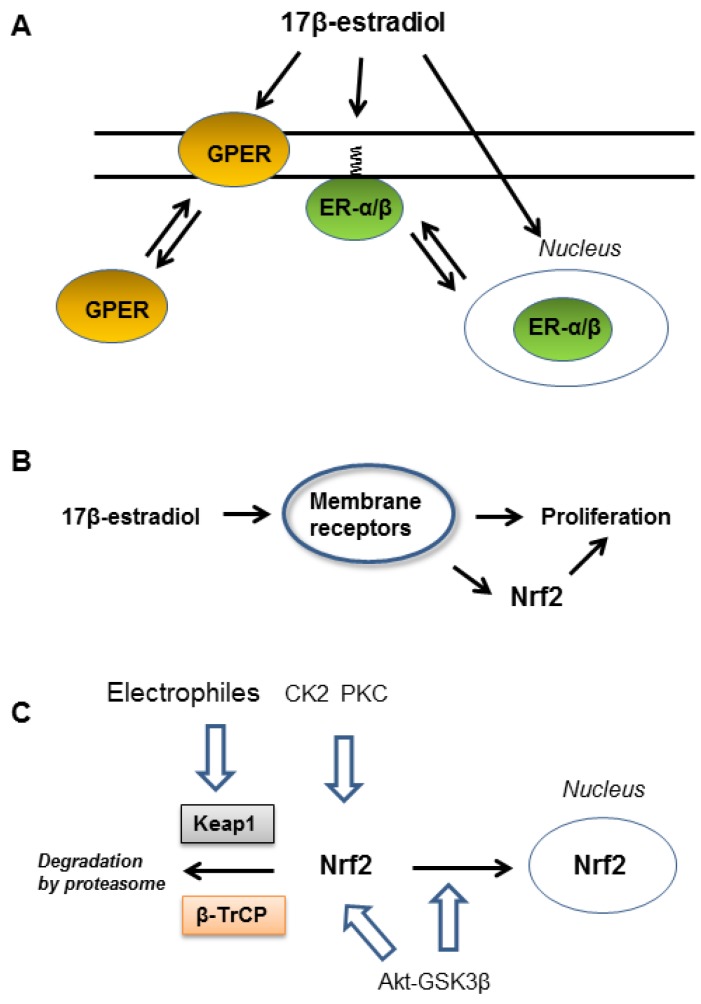
17β-estradiol activates nuclear factor-E2-related factor 2 (Nrf2) and enhances cell proliferation. (**A**) Part of G protein-coupled estrogen receptor (GPER) and estrogen receptors α and β (ER-α/β) are associated with plasma membrane and transduce mitogenic signals elicited by 17β-estradiol. (**B**) 17β-Estradiol acting through membrane-associated estrogen receptors stimulates proliferation of human breast cancer MCF-7 and other cells. Rapid Nrf2 activation by 17β-estradiol enhances proliferation. (**C**) Nrf2 is unstable due to Kelch-like ECH-associated protein 1 (Keap1)- and β-TrCP (transducing repeat-containing protein)-mediated ubiquitylation and following degradation by proteasome. Rapid activation of Nrf2 is regulated by both stabilization and facilitation of nuclear import of Nrf2. Electrophiles react with Keap1 leading to stabilization of Nrf2. Protein kinase C (PKC) and casein kinase 2 (CK2) phosphorylate/stabilize Nrf2. Glycogen synthase kinase 3β (GSK3β) phosphorylates Nrf2 to induce β-TrCP-mediated degradation and also suppresses nuclear import of Nrf2. Akt (protein kinase B) phosphorylates/inactivates GSK3β to enhance Nrf2 activation.

Estrogens have long been known as important regulators of the female reproductive functions. However, it is now commonly accepted that estrogens are locally synthesized and play important roles in the cardiovascular protection, bone preservation, and neuroprotection in both female and male (reviewed in [1,2,3]). Estrogens transduce signals through two signaling pathways via nuclear receptors estrogen receptor (ER)-α and ER-β, and plasma membrane-associated receptors [4,5,6]. The G-protein coupled estrogen receptor GPER/GPR30 is a 7-transmembrane receptor implicated in rapid estrogen signaling [7,8,9]. However, estrogen signaling is complex because ER-α and ER-β receptors can be palmitoylated to localize the cell membrane [5,10] and ER-α has three isoforms termed ER-α66, ER-α46 and ER-α36, derived from different promotor usage and alternative splicing [6]. Additionally, GPER undergoes constitutive retrograde trafficking from the plasma membrane to the endoplasmic reticulum and its surface expression is limited and controlled by intrinsic factors [7]. These estrogen receptors are expressed in cell type-specific manners increasing variation of signaling pathways as reviewed in [11,12,13,14] (Figure 1A).

One of the biological functions of estrogen is stimulation of cell proliferation. ER-α is the major driver of breast cancer and multiple ER targeting drugs are routinely used clinically to treat patients with ER positive breast cancer [15,16,17]. Accumulating evidence indicates non-genomic rapid estrogen signaling via membrane-associated estrogen receptors such as ER-α36 and GPER play important roles in mitogenic estrogen signaling [6,7]. Transactivation of epidermal growth factor receptor (EGFR) via membrane-associated estrogen receptors plays a key role for the promotion of cell proliferation [6,7]. A rapid cellular response following mitogenic stimulation is activation of transcription factor nuclear factor-E2-related factor 2 (Nrf2), which regulates the expression of a large number of genes, including detoxification enzymes, antioxidant proteins and glutathione (GSH) synthesis system via the antioxidant responsive element (ARE) in a cell type specific manner [18,19,20]. Additionally, Nrf2 upregulates metabolic genes involved in cell proliferation and differentiation [21]. Nrf2 promotes proliferation of various cancer cells and enhances resistance to anticancer drugs [22,23,24,25]. For example, stimulation of human breast cancer MCF-7 cells with nM levels of 17β-estradiol rapidly activates Nrf2 via the PI3K dependent manner [26]. However, precise signaling cascades downstream of estrogen receptors leading to rapid activation of Nrf2 are currently not clear (Figure 1B).

Rapid Nrf2 activation can be induced by both stabilization of Nrf2 and enhanced nuclear import of Nrf2 protein. Nrf2 protein is unstable under normal conditions because the cytosolic Nrf2 binding protein Kelch-like ECH-associated protein 1 (Keap1) facilitates degradation of Nrf2 by proteasome [27,28,29]. It is well known that electrophilic compounds chemically react with sulfhydryl residues in Keap1 and inhibit degradation of Nrf2 [27,28,29]. In addition to Keap1, another ubiquitin ligase adaptor β-transducin repeat-containing protein (β-TrCP) also destabilizes Nrf2 when glycogen synthase kinase 3β (GSK3β) phosphorylates serine residues in Nrf2 [30,31]. It is also proposed that GSK3β suppresses nuclear import of Nrf2 [32,33]. High glucose or diabetic conditions accompanies upregulation of GSK3β activity and facilitates degradation of Nrf2 [34]. Therefore, inhibition of GSK3β by protein kinase Akt (protein kinase B) secures activation of Nrf2 [30,33]. Additionally, some protein kinases such as protein kinase C (PKC) [35] and casein kinase 2 (CK2) [36,37,38] directly phosphorylate and protect Nrf2 from Keap1-dependent degradation (Figure 1C).

This review highlights the signaling pathways leading to rapid activation of Nrf2 by 17β-estradiol and some estrogen mimetics via membrane-associated estrogen receptors. Nrf2 activation via hormone-receptor signaling cascades differs from that induced by toxic electrophiles which directly target Keap1. We recently proposed that neurotrophins can activate Nrf2 by a mechanism dependent on the common neurotrophin receptor p75^NTR (neurotrophin receptor)^ [39,40,41]. This hypothesis is based on the facts that nerve growth factor activates CK2 by a p75^NTR^-depndent manner [42] and that CK2 directly phosphorylates Nrf2 to stabilize/activate it [36,37]. Notably, stimulation of p75^NTR^ induces generation of the lipid signal mediator ceramide via activation of neutral sphingomyelinase (nSMase) [43]. As ceramide activates PKCζ at low concentrations [44] and PKCζ can phosphorylate/activate CK2 [45], we hypothesized that neurotrophins activate Nrf2 via p75^NTR^-ceramide-PKCζ-CK2 pathway [39], although mechanism of nSMase activation via p75^NTR^ is currently not known. We here propose novel signaling pathways downstream of membrane-associated estrogen receptors leading to Nrf2 activation with a special interest in the regulation of proliferation of estrogen-dependent breast cancer cell MCF-7.

## 2. Role of ER-α36 in EGFR-Dependent Activation of Nrf2

ER-α36 is widely expressed in estrogen sensitive cancer cells, and high expression levels correlates with a poor survival prognosis for breast cancer patients [46]. MCF-7 cells express ER-α66 as the major ER-α, but also express ER-α isoforms, ER-α46 and ER-α36 [6]. ER-α36 differs from the ER-α66 by lacking N- and C-terminal transcriptional activation domains but retaining the central DNA-binding and dimerization domains, and partial ligand-binding domains. It also possesses an extra, unique 27 amino acid domain to replace the last 138 amino acids of the ER-α66 [6]. All three ER-α isoforms contain the cysteine residue in the ligand binding domain for *S*-palmitoylation, which is a reversible post-translational lipid modification important for membrane anchoring [47].

ER-α36 localizes in the cytoplasm and at the plasma membrane, mediating rapid estrogen signaling in human breast cancer cells [6] and plays a key role in estrogen-dependent proliferation [48]. Post-translational palmitoylation is suspected to play a crucial role in anchoring ER-α36 to the cell membrane and interaction with caveolin-1 [10]. Activation of ER-α36 leads to activation of phospholipase D and PKC in breast cancer cells [49]. Stimulation of membrane-associated estrogen receptors accompanies transactivation of epidermal growth factor receptor (EGFR), inducing rapid kinase cascades that influence both transcriptional and non-transcriptional actions of estrogen [50].

**Figure 2:**
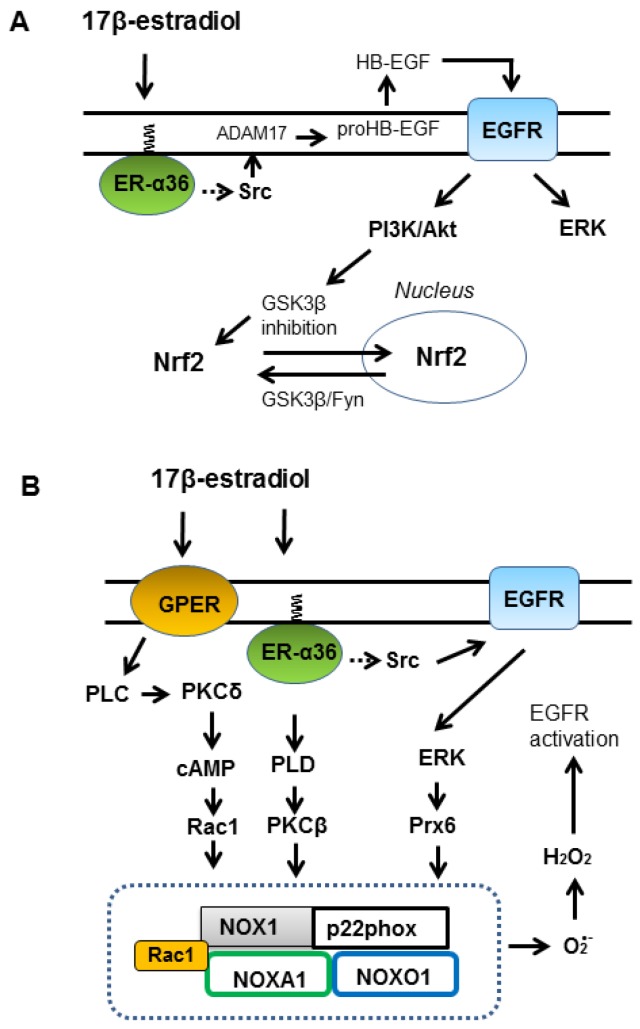
Activation of epidermal growth factor receptor (EGFR) and NADPH oxidase 1 via membrane-associated estrogen receptors. (**A**) Stimulation of ER-α36 induces transactivation of EGF receptor (EGFR) leading to activation of Ras/RAF/MEK/ERK and Ras/PI3K/PTEN/Akt pathways. Activated Akt inhibits GSK3β and enhances nuclear import of Nrf2 and stability of Nrf2. HB-EGF (heparin-binding EGF-like growth factor), ADAM17, PLC (phospholipase C), PLD (phospholipase D), Prx6 (peroxiredoxin 6), NOXA1, NOXO1. (**B**) GPER-mediated signaling cooperate with ER-α36-EFGR signaling leading to activation of NADPH oxidase 1 (NOX1), which generates superoxide. Superoxide is metabolized to H_2_O_2_, which enhances EGFR signaling. Activation of Rho family GTPase Rac1, PKCβ and ERK can induce NOX1 activation.

EGFR is expressed in various cell types and plays a key role in the regulation of cell proliferation, survival and differentiation. In pathological settings, mostly in lung and breast cancer and in glioblastoma, EGFR is overexpressed or mutated [51,52,53]. Activation of EGFR can be caused by the release of heparin-binding EGF-like growth factor (HB-EGF) from membrane-anchored form (proHB-EGF) by a metalloprotease ADAM17. Although intracellular Ca^2+^ elevation is required for the activation of tyrosine kinase Src which lead to activation of ADAM17 [54], precise mechanism of Src activation is not known. EGFR signaling activates Ras/RAF/MEK/ERK and Ras/PI3K/PTEN/Akt pathways [55,56]. Notably, treatment of MCF-7 cells with 1 nM 17β-estradiol induced rapid association of ER-α36 with Src at the plasma membrane and activation of ERK in 5 min [57], and activation/phosphorylation of Akt and inhibition/phosphorylation of GSK3β in 15 min [26]. As GSK3β destabilizes Nrf2 and suppresses Nrf2 nuclear import, activation of Akt plays a key role in the activation of Nrf2-ARE-mediated target gene expression [30,31,32,33]. Wu et al. [26] showed that a PI3K-specific inhibitor wortmannin significantly inhibited Nrf2 activation by 17β-estradiol in MCF-7 cells [26]. These studies suggest EGFR-PI3K-Akt-GSK3β signaling downstream of ER-α36 largely contributes Nrf2 nuclear import and activation of gene transcription via ARE elements at physiological concentrations of 17β-estradiol (Figure 2A). However, Wu et al. [26] also showed that a kinase inhibitor LY294002, which inhibits both PI3K and CK2, also inhibited Nrf2 activation by 17β-estradiol in MCF-7 cells more efficiently than wortmannin [26].

ER-α36 is expressed in rat and human brain especially high levels in cerebral cortex and plays a role in protection of neurons under oxygen and glucose deprivation [58]. Estrogen synthetic enzyme aromatase is expressed in neurons under basal conditions and is upregulated in reactive astrocytes after injury [59]. Zhu et al. [60] showed intravitreal injection of 17β-estradiol induced rapid activation of Nrf2 in retina’s cell layers and protected the tissue from light-induced degeneration [60]. After the 17β-estradiol treatment, Nrf2 protein levels significantly increased in 4 h and peaked in 12 h. The kinase inhibitor LY294002 effectively blocked Nrf2 activation in retina’s cell layers [60].

## 3. Functions of Membrane-Associated GPER

Cloned GPER cDNA predicts GPER is a 375 amino acid protein with the structural signature of the Class A Rhodopsin family 7-transmembrane receptor with about 30% amino acid identity to angiotensin II (AngII) and interleukin 8 receptors [9]. Compared to ER-α36, GPER is expressed ubiquitously and has now become recognized as the major mediator of estrogen’s rapid cellular effects throughout the body. It is not involved in reproduction but GPER deficiency results in multiple physiological alterations including obesity, cardiovascular dysfunction, insulin resistance and glucose intolerance (reviewed in [61]). Activation of GPER can mediate multiple salutary effects on the cardiovascular system and GPER-selective agonist G1 can be considered a non-feminizing estrogenic compound and thus of potential therapeutic use in both women and men.

GPER associates with Gα_s_ protein and activates phospholipase C (PLC) to catalyze the hydrolysis of membrane-bound phosphatidylinositol 4,5-bisphosphate (PIP_2_) into inositol 1,4,5-triphosphate (IP_3_)and diacylglycerol (DAG) [62]. DAG activates PKCδ to augment adenylyl cyclase activity generating cAMP [45]. Additionally, GPER has two notable functions, which may not necessarily beneficial for health. First, it regulates Ca^2+^ signaling via interaction with plasma membrane Ca^2+^-ATPase (PMCA) [63]. PMCA is essential for removal of cytoplasmic Ca^2+^ and for shaping the time courses of Ca^2+^-dependent activity. The authors show that GPER physically associates with PMCA and suppresses PMCA activity. GPER agonist G1 further inhibits PMCA activity through tyrosine phosphorylation of the pump resulting increase in intracellular Ca^2+^ levels. Second, GPER enhances expression of NADPH oxidase 1 (NOX1) [64,65]. NOX1 generates superoxide which is instantly metabolized to hydrogen peroxide (H_2_O_2_) by superoxide dismutase. Meyer et al. [64] observed that genetic ablation of GPER in mice prevented cardiovascular pathologies associated with aging by reducing superoxide formation through downregulation of NOX1 expression. Blocking GPER activity with specific antagonist G36 decreased NOX1 abundance and decreased superoxide production and arterial hypertension in mice chronically infused with AngII (0.7 mg/kg body weight/day) [64,65].

In addition to vascular cells [66,67], NOX1 is expressed various cells including neurons and cancer cells [68]. Activation of NOX1 and generated superoxide and H_2_O_2_ serve as co-stimulatory signals for cell proliferation in many normal and cancer cells [69,70,71,72,73]. NOX1-derived ROS promoted phosphorylation of ERK1/2 and expression of cyclin D1 and Fos family genes during the immediate early gene response [74]. As important consequences of GPER activation are the regulation of cell growth, migration, and apoptotic cell death [75], GPER may contribute NOX1 activation in addition to upregulation of NOX1 expression.

## 4. Cooperation of ER-α36 and GPER in NADPH Oxidase 1 Activation

Activation of NOX1 requires association with its regulatory components p22phox, NOX organizer 1 (NOXO1), NOX activator 1 (NOXA1) and Rho family small GTPase Rac1 [68]. Choi et al. [76] ectopically over-expressed AngII type 1 receptor (AT_1_R), NOX1, NOXO1 and NOXA1 using virus vectors into HEK293 and CHO cells and characterized mechanism and kinetics of NOX1 activation by AngII [76]. The activation of NOX1 by AngII was very fast and the production of superoxide peaked at around 10 min but decayed faster. The earliest response of AngII-AT_1_R signaling is Gα_q_-mediated activation of phospholipase-β, which is followed by Ca^2+^ mobilization and activation of PKC, and G protein-dependent and -independent activation of Src family kinases [77]. The authors observed that extracellularly added EGF also induces NOX1 activation in the model cells, which had additionally expressed EGFR, with a similar kinetics but generated superoxide was lower compared to that by AngII-AT_1_R-NOX1 under the experimental conditions [76]. This result suggests that once EGFR is activated it will enhance NOX1 activation. Notably, single addition of PKC agonist phorbol 12-myristate 13-acetate at 0.3 μM induced NOX1 activation and superoxide generation in the model cells at similar extent as that by 1 μM AngII [76]. Notably, activation of Src is required for AngII induced NOX1 activation, and inhibition of PI3K by wortmannin or LY294002 attenuated both Rac activation and ROS generation in vascular endothelial cells [78].

These studies suggest that ER-α36 and GPER respectively facilitate NOX1 activation. NOX1 phosphorylation at Thr429 by PKCβ facilitates the association of NOX1 with the NOXA1 in vascular smooth muscle cells [79] and GPER-mediates increases in cAMP to activate Rac1 via cAMP dependent Epac1/Rap1 signaling [80]. Moreover, in tumor colon epithelial cells, activated Rac1 interacts with NOXA1 to enhance NOX1 activity [81]. GPER-mediated Ca^2+^ mobilization may activate PKC and contribute in NOX1 activation like the phorbol ester (Figure 2B). However, currently there is no report showing 17β-estradiol at low levels activates NOX1 in MCF-7 or other cells. However, Lei et al. [82] showed that stimulation of MCF-7 cells with bisphenol F (a estrogenic pollutant, 10–100 nM) induced rapid but marginal increases in both intracellular Ca^2+^ and ROS levels in an ER-α and GPER dependent manner [82]. Bisphenol F is one of the alternatives to an estradiol mimetic bisphenol A and induces proliferation of MCF-7 cells similar to 17β-estradiol [83].

GPER forms a complex with ER-α36 and also with p65 component of NF-κB, acting as suppressor of NF-κB in human primary monocytes [84]. Additionally, stimulation of GPER by 1 nM 17β-estradiol induces rapid gene expression of ER-α36 by an AP-1 dependent manner in SK-BR-3 breast cancer cells [85] and by PKA/CREB pathway in seminoma-like TCam-2 cells [86]. In these cancer cell lines, upregulation of ER-α36 is required for cell proliferation. Expression of EGFR is also upregulated in TCam-2 cells by the 17β-estradiol treatment [86]. Interestingly, GPER-EGFR-ERK-c-Fos-AP-1 signaling also upregulates GPER expression in MCF-7 cells [83] and spermatogonial GC-1 cells [87]. These studies suggest existence of a functional cooperation between GPER, ER-α36, EGFR and NOX1 in supporting cell proliferation following stimulation with 17β-estradiol or estrogen mimetics (Figure 2B).

Once NOX1 is activated, superoxide and H_2_O_2_ will be generated. NOX1 transfers an electron from intracellular NADPH across the cell membrane to molecular oxygen to generate superoxide in the extracellular space or within a signaling endosome if the NOX1 is endocytosed with the membrane receptors [88]. Importantly, extracellular or intra-endosomal superoxide can penetrate cell membranes through a chloride channel-3 (CIC-3) [88]. Cellular superoxide dismutase converts superoxide to H_2_O_2_, which can traverse the cell membrane through aquaporin channels [89]. Therefore, both superoxide and H_2_O_2_ may interact with specific cellular targets to initiate intracellular signaling. Recent studies show that 17β-estradiol upregulates expression of CIC-3 channels by an ER-α-dependent manner in MCF-7 cells [89] and that the CIC-3 channel activity contributes proliferation of MCF-7 and MDA-MB-231 breast cancer cells [90].

As low levels of H_2_O_2_ inhibit activity of protein tyrosine phosphatases which result in the activation of receptor tyrosine kinase [91], activation of NOX1 will contribute to maintain/enhance EGFR signaling. At the same time EGFR activation may help NOX1 activation as demonstrated in the model cells [76]. Association of NOXO1 to plasma membrane requires the presence of phosphatidylinositol lipids [92]. It is produced by calcium-independent phospholipase A_2_ or peroxiredoxin 6, which is activated/phosphorylated by ERK and p38 [93]. Thus, once NOX1 and EFGR are activated, ER-α36-GPER-EGFR-NOX1 seems to form a positive feedback loop to further enhance EGFR-mediated signaling cascade (Figure 2B). However, we think superoxide generated by NOX1 plays another important function to support EGFR-mediated kinase cascade.

## 5. Superoxide Dependent Activation of Neutral Sphingomyelinase 2

We suggest that GPER-ER-α36-NOX1 signaling could rapidly induce activation of neutral sphingomyelinase 2 (nSMase2), which hydrolyzes sphingomyelin to generate ceramide. Ceramide is a pleiotropic signal mediator of cellular responses including differentiation, proliferation, cell cycle arrest and apoptosis [94].

A notable characteristic of nSMase2 is that enzyme activity can be reversibly inhibited by GSH. Liu and Hannun [95] partially purified nSMase2 from MCF-7 cells and observed that reduced GSH at 1 to 3 mM almost linearly inhibited the activity from about 0 to 93% with a 50% inhibition at around 1.7 mM. These concentrations of GSH have physiological relevance as cultured cells have a few mM GSH depending on cell types and culture conditions [96]. Interestingly, the authors showed that dilution of the pre-incubated medium containing the enzyme plus GSH induced rapid recovery of the enzyme activity suggesting the inhibition by GSH is reversible. They also showed that reducing agents, dithiothreitol and β-mercaptoethanol, neither inhibit nSMase activity nor disturb the inhibition by GSH, suggesting reduction of disulfide(s) may not play a role in the GSH-mediated enzyme inhibition. These results clearly suggest that nSMase2 has a low affinity GSH binding site and that GSH acts as a ligand to inhibit nSMase2 activity by a non-covalent and reversible manner. The inhibition of nSMase2 by GSH may be a feedback response to maintain cellular GSH levels as low levels of ceramide could activate Nrf2 leading to upregulation of GSH synthesis via expression of the cystine transporter and γ-glutamyl-cysteine synthetase [39,40,41]. The idea that GSH acts as a non-covalent ligand is already known in case of feedback inhibition by GSH of γ-glutamyl-cysteine synthetase, the rate limiting enzyme for GSH synthesis [97].

**Figure 3:**
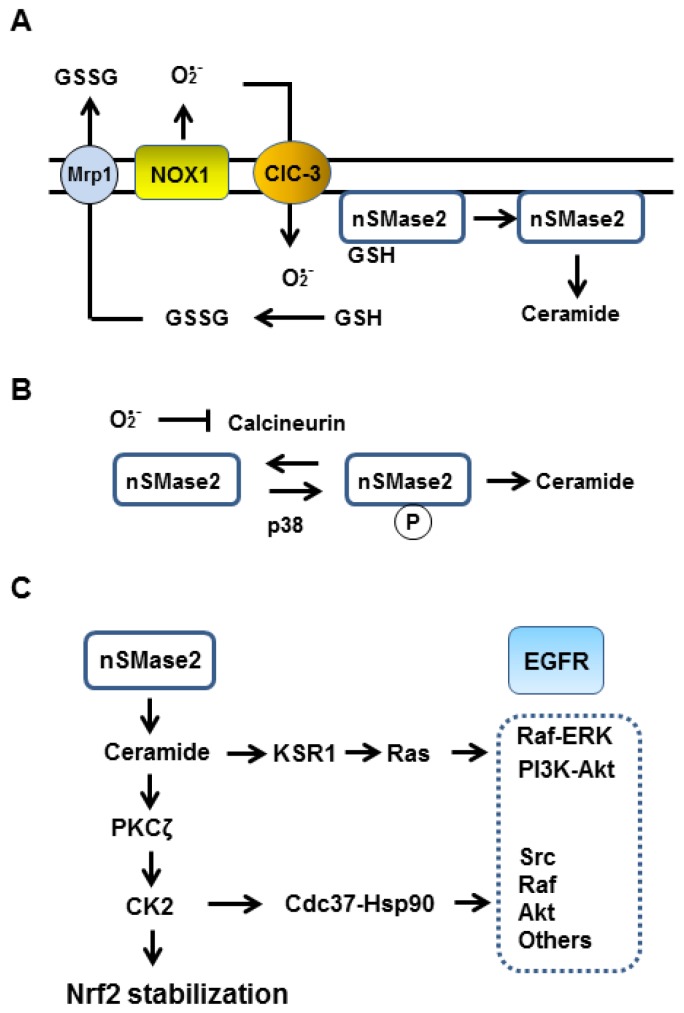
Role of neutral sphingomyelinase-2 (nSMase2) in Nrf2 activation and transactivation of EGFR. (**A**) NOX1 extracellularly generates superoxide, which can pass cell membrane through chloride channel-3 (CIC-3) to oxidize cellular glutathione (GSH) to GSSG. As GSSG can rapidly be exported from cells via multidrug resistant protein 1 (Mrp1), activation of NOX1 leads to temporal decrease in cellular GSH levels. GSH non-covalently binds neutral sphingomyelinase-2 (nSMase2) to inhibit the enzyme activity. Decrease in GSH induces nSMase2 activation and generation of lipid signaling molecule ceramide. (**B**) Superoxide inactivates calcineurin, which is bound to nSMase2 to inhibit from activation by p38. (**C**) Ceramide plays a crucial role for the transactivation of EGFR. Ceramide is required for KSR1 (kinase suppressor of Ras 1)-mediated activation of Ras, and CK2 plays an important role in the activation of Cdc37-Hsp90 molecular chaperone which mediates activation of signaling protein kinases, including Src, Raf and Akt. Ceramide-PKCζ-CK2 axis stabilizes/activates Nrf2 [39].

Taken together, nSMase2 activity seems to be suppressed in cells under normal conditions with high GSH and that the enzyme activation requires downregulation of cellular GSH. Notably, superoxide but not H_2_O_2_ rapidly and effectively oxidizes GSH to generate disulfide GSSG [98], which can be exported from cells [99]. Importantly, the complete or significant depletion of whole cellular GSH is not required for the superoxide dependent transient activation of nSMase2 as membrane estrogen receptors, NOX1, nSMase2 and EGFR may be closely located in the membrane and NOX1-derived superoxide would rapidly down-regulate GSH in a cellular micro-compartment or micro-environment and facilitate activation of closely located nSMase2 (Figure 3A).

The activation of nSMase2 is also modulated by protein kinases. PKCδ mediates translocation of nSMase2 from the Golgi to the plasma membrane [100]. Phosphorylation by p38 mitogen-activated protein kinase is important for the activation of nSMase2 in cigarette smoke-treated human airway epithelial cells [101] and in rat hippocampal astrocytes following cerebral ischemia [102]. Filosto et al. [103] showed that calcineurin (protein phosphatase 2B) directly associates with nSMase2 and suppresses phosphorylation/activation of nSMase2. Calcineurin is a serine/threonine phosphatase involved in a wide range of cellular responses to calcium mobilizing signals. Calcineurin has a Fe-Zn active center which becomes sensitive to superoxide in its calcium-activated conformation [104], and copper/zinc superoxide dismutase protects calcineurin [105,106]. Therefore, it is possible that p38 may phosphorylate/activate nSMase2 when superoxide is generated to inactivate calcineurin (Figure 3B). As Src can activate p38 [107] and PKCδ [108], once Src is stably activated, nSMase2 activity may be enhanced or maintained while superoxide is generated. Taken together, generation of superoxide could cause activation of nSMase2 in at least two ways, via oxidation of GSH and inactivation of calcineurin (Figure 3A,B).

In fact, it was shown that superoxide can activate nSMase in cultured cells [109,110]. Sawada et al. [109] examined effects of DNA damaging agent etoposide on ceramide-dependent apoptosis of human glioma cells and found long lasting nSMase activation which was dependent on prolonged generation of superoxide rather than H_2_O_2_. In case of amyloid-β-induced neuronal death, nSMase activation occurred by a NADPH oxidase-superoxide-H_2_O_2_ dependent manner [110]. These cases suggest that superoxide is involved in nSMase activation and that long lasting ceramide generation or higher levels of ceramide induces cell apoptosis rather than protection. Notably, ceramide effectively activates PKCζ only at concentrations between 1 and 60 nM, and higher concentrations above 100 nM hardly enhance autophosphorylation/activation of PKCζ [44]. Therefore, mild and temporal increases in ceramide levels via NOX1-nSMase2 axis can induce PKC-ζ-CK2-mediated phosphorylation/stabilization of Nrf2 (Figure 3C).

## 6. Ceramide Activates EGFR-Mediated Signaling Kinase Cascade

Ceramide generation by nSMase2 seems to be required for the EGFR mediated signaling cascade. This idea comes from the two facts; (i) Ras scaffolding protein KSR1 is a ceramide-binding protein requiring low levels of ceramide for membrane translocation to trigger the Ras signaling via EGFR [111,112] and (ii) nSMase inhibitor GW4869 thoroughly abrogates activation of ERK and p38 MAP kinases MAPKs in rat glioma C6 cells stimulated by phytochemicals, pent-acetyl geniposide [113] or caffeic acid phenethyl ester (CAPE) [114]. These compounds respectively induce rapid activation of nSMase (peaking at 15 min) and ERK and p38 phosphorylation/activation within 30 min in C6 cells, noting these authors suggested ceramide generation is required for activation of MAPKs [113,114].

Geniposide, a major iridoid component of *Gardenia* fruit, is an agonist for G-protein coupled glucagon-like peptide-1 (GLP-1) receptor [115] and GLP-1 is known to transactivate EGFR [116]. Therefore, GLP-1 receptor functions like GPER in transactivation of EGFR. GLP-1 induces pancreatic β-cell proliferation and induces Nrf2 activation [117]. Geniposide and its structurally-related compounds are known to activate Nrf2 [118,119]. Peng et al. [113] showed that treatment of rat glioma C6 cells with pent-acetyl geniposide induced rapid and temporal activation of nSMase with intracellular ceramide levels increasing three to four fold at 2 to 3 h. Under the experimental conditions, these authors observed a rapid and temporal decrease in intracellular total GSH levels in C6 cells. The GSH levels decreased by about 30% in 0.5 to 1 h, thereafter the GSH levels gradually recovered in 2–3 h. They did not detect any peroxidized lipid metabolites in the cells, suggesting limited oxidative cell damage occurred under the experimental conditions. Although the authors did not measure superoxide generation in the C6 cells in the early time phase of pent-acetyl geniposide treatment, we speculate that superoxide was generated to oxidize GSH to GSSG, which was rapidly exported from cells via multidrug resistance protein Mrp1 [99] leading to temporal decrease in cellular GSH.

CAPE is an active ingredient of coffee, vegetables and beehive propolis and shown to induce Nrf2 activation in renal epithelial cells [120] and in mice brain [121]. As CAPE has a weak affinity for estrogen receptors ERα and ERβ, it may also interact with membrane-associated estrogen receptors [122]. Although these authors used the compounds at a high concentration (50 μM), the results suggest a possibility that GPER/GLP-1R-NOX1-nSMase2-ceramide-KSR1 signaling is necessary for Ras-PI3K/ERK signaling downstream of Src-activated EGFR in C6 cells (Figure 3C).

In addition to ceramide-KSR1 signaling, ceramide-PKCζ-CK2 signaling also plays a key role in enhancing kinase signaling via interaction of CK2 with molecular chaperone Cdc37 [123]. Activation of CK2 drastically changes cell metabolism [124] partly through interaction with Cdc37 [125]. Cdc37 is known as a cochaperone of Hsp90 interacting with many signaling protein kinases including Src, Raf-1 and Akt [123,125,126]. Miyake and Nishida [123] showed that CK2 associates with Cdc37 and phosphorylates Ser13 leading to increase in its chaperone activity resulting in activation of associated kinases. Therefore, nSMase-ceramide-CK2 signaling contributes to activation of Src to enhance ADAM17 mediated EGFR activation and in kinase signaling cascade downstream of EGFR (Figure 3C).

## 7. Conclusions and Future Perspectives

**Figure 4:**
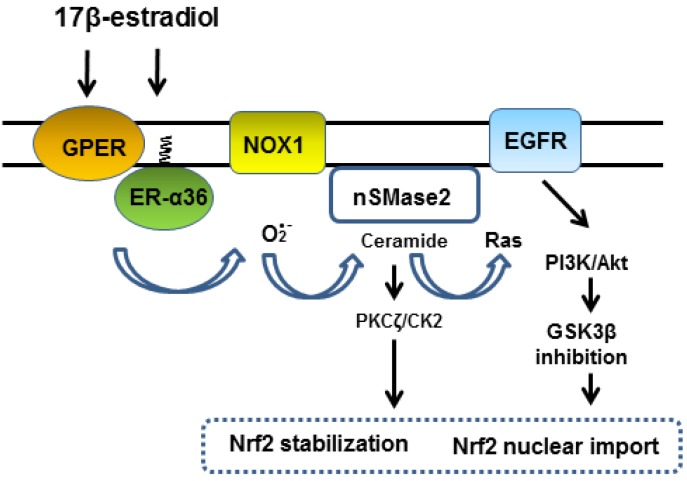
Summary of signal transduction mediated by membrane-associated estrogen receptors leading to activation of Nrf2. 17β-Estradiol via GPER and ER-α36 receptors initially activates Src and NOX1 to generate superoxide, which in turn activates nSMase2 to generate the signal mediator ceramide. Ceramide plays a key role for the activation of CK2 and the EGFR-mediated kinase cascade. Direct modification of Nrf2 by CK2 and enhanced nuclear import of Nrf2 by Akt/GSK3β achieve rapid and effective Nrf2-ARE mediated gene expression. Inhibition of GSK3β also suppresses β-TrCP dependent Nrf2 degradation.

We propose that the stimulation of membrane-associated estrogen receptors initially activates NOX1. NOX1-derived superoxide activates nSMase2 to generate ceramide which subsequently activates Ras and PKCζ-CK2 axis. The CK2-Cdc37-Hsp90 axis supports EGFR-mediated signaling kinase cascade. Activation of Nrf2 depends on both PI3K-Akt-GSK3β and PKCζ-CK2 signaling pathways (Figure 4). As PI3K seems to be required for NOX1 activation [78], the kinase inhibitor LY294002, which inhibits both PI3K and CK2 activities, could significantly suppress Nrf2-ARE signaling as was observed in MCF-7 cells following treatment with 10 nM 17β-estradiol [26].

The importance of superoxide in Nrf2 activation has been demonstrated in vascular endothelial cells exposed to vasoprotective unidirectional laminar shear stress [127]. Fluid shear stress regulates endothelial cell function but the precise mechanism involved in mechanotransduction remains unclear. Czarny et al. [128,129] showed that nSMase is concentrated at the endothelial cell surface in caveolae and is activated to produce ceramide in an acute and transient manner in response to increased flow rate and pressure in rat lung vasculature. We speculate that the shear stress activates NOX1 which subsequently induces nSMase2-ceramide signaling leading to Nrf2 activation. The authors showed that extracellularly supplemented cell-permeable ceramide analogs induced activation of Src and ERK kinases, and also the Akt-dependent endothelial nitric oxide synthase (eNOS) pathway [129]. These studies suggest that an unidentified sensor for mechanical stress induces rapid activation of NOX1-nSMase2-ceramide axis, which then leads to PKCζ-CK2 and PI3K-Akt signaling in vascular endothelial cells by similar signaling pathways shown in Figure 3; Figure 4. It is noted that the estrogen mimetic isoflavone Equol via membrane-associated receptors rapidly activates eNOS/Hsp90 by an ERK and Akt dependent manner in human endothelial cells [130]. Taken together, NOX1, nSMase2 and EGFR seems to compose a common platform in membrane caveolae to mediate various receptor-mediated rapid signaling cascades. Probably membrane-associated estrogen receptors share this signaling platform to supplement or boost various signaling pathways including mechanotransduction in vascular endothelial cells and GLP-1R signaling in β-cell. This idea could explain why estrogen signaling is important in the cardiovascular protection, bone preservation, neuroprotection, metabolism and proliferation of various cell types.

Rapid activation of Nrf2 by low levels of 17β-estradiol will favor proliferation of MCF-7 cancer cells [26] by facilitating metabolic reprogramming through upregulation of enzymes for glucose metabolism and at the same time suppresses generation of ROS to protect cells from oxidative damages. The membrane-associated estrogen receptors are targets for phytoestrogens and endocrine-disrupting substances [8,131]. In addition to the phytoestrogen genistein, chemically synthesized compounds bisphenol A and nonylphenol have high affinities for ER-α, ER-β and GPER [8,131]. Cadmium chloride activates GPER-mediated signaling at concentrations 5 to 500 nM and promotes cell proliferation like 17β-estradiol in human breast cancer, lung adenocarcinoma and thyroid cancer cells [132,133,134]. These compounds may increase cancer risks partly through promotion of cell proliferation [135,136]. Further studies on NOX1-nSMase2-ceramide axis will help to find novel strategies for hormone replacement and cancer therapies.

## Abbreviations

Ang II	angiotensin II
ARE	antioxidant responsive element
AT_1_R	angiotensin II type 1 receptor
CIC-3	chloride channel 3
CK2	casein kinase 2
DAG	diacylglycerol
EGFR	epidermal growth factor receptor
eNOS	endothelial nitric oxide synthase
GLP-1	G-protein coupled glucagon-like peptide-1
GPER	G-protein coupled estrogen receptor
GSK3β	glycogen synthase 3 β
Keap1	Kelch-like ECH-associated protein 1
Mrp1	multidrug resistance protein 1
NOXA1	NOX activator 1
NOX1	NADPH oxidase 1
NOXO1	NOX organizer 1
Nrf2	nuclear factor-E2-related factor 2
nSMase2	neutral sphingomyelinase 2
PLC	phospholipase C
PMCA	plasma membrane Ca^2+^-ATPase
